# Expression and functional studies of genes involved in transport and metabolism of glycerol in *Pachysolen tannophilus*

**DOI:** 10.1186/1475-2859-12-27

**Published:** 2013-03-21

**Authors:** Xiaoying Liu, Uffe Hasbro Mortensen, Mhairi Workman

**Affiliations:** 1 Department of Systems Biology, Building 223, Søltofts Plads, Technical University of Denmark, Lyngby 2800 Kgs, Denmark

**Keywords:** *P. tannophilus*, Glycerol, Transport, *S. cerevisiae*

## Abstract

**Background:**

*Pachysolen tannophilus* is a non-conventional yeast, which can metabolize many of the carbon sources found in low cost feedstocks including glycerol and xylose. The xylose utilisation pathways have been extensively studied in this organism. However, the mechanism behind glycerol metabolism is poorly understood. Using the recently published genome sequence of *P. tannophilus* CBS4044, we searched for genes with functions in glycerol transport and metabolism by performing a BLAST search using the sequences of the relevant genes from *Saccharomyces cerevisiae* as queries.

**Results:**

Quantitative real-time PCR was performed to unveil the expression patterns of these genes during growth of *P. tannophilus* on glycerol and glucose as sole carbon sources. The genes predicted to be involved in glycerol transport in *P. tannophilus* were expressed in *S. cerevisiae* to validate their function. The *S. cerevisiae* strains transformed with heterologous genes showed improved growth and glycerol consumption rates with glycerol as the sole carbon source.

**Conclusions:**

*P. tannophilus* has characteristics relevant for a microbial cell factory to be applied in a biorefinery setting, *i.e.* its ability to utilise the carbon sources such as xylose and glycerol. However, the strain is not currently amenable to genetic modification and transformation. Heterologous expression of the glycerol transporters from *P. tannophilus*, which has a relatively high growth rate on glycerol, could be used as an approach for improving the efficiency of glycerol assimilation in other well characterized and applied cell factories such as *S. cerevisiae*.

## Background

*Pachysolen tannophilus* is known for its ability to ferment D-xylose, one of the major components of hemicellulose plant residues, to ethanol [1]. However, *P. tannophilus* has also been shown to be capable of converting crude glycerol to ethanol under microaerobic conditions [2]. This ability is interesting since glycerol, a by-product of biodiesel production, has also been considered as a potential alternative carbon source for industrial bioprocesses due to the recent dramatic increase in production of biodiesel.

The whole genome of *P. tannophilus* CBS4044 has been sequenced [3], and with this, the possibility for understanding and exploiting glycerol transport in this yeast has arisen. A number of studies have investigated glycerol transport, consumption and production by different types of yeast [4-6], but the glycerol transport and metabolic pathways in *P. tannophilus* have not been studied so far. *P. tannophilus* has a relatively high growth rate on glycerol (0.3 h^-1^) [2], but improvement of the strain for industrial applications through engineering the metabolism is hampered due to the lack of tools for genetic modification. However, with glycerol becoming an increasingly abundant substrate, it is highly relevant to characterize strains which are capable of its utilization, and consider the possibility of expressing genes from these strains in industrial cell factories for which genetic engineering strategies are already well defined.

Glycerol transport is the first barrier to glycerol utilization in a microbial cell. In *S. cerevisiae*, glycerol enters the cell by two different mechanisms: a low affinity transport system (facilitated diffusion) and a high affinity proton symport system (active transport) [7]. When glucose is present, glycerol diffuses into the cells through a glycerol channel by facilitated transport, a process dependent on the *FPS1* gene [8,9]. The physiological role of the facilitator Fps1p in *S. cerevisiae* has been described to be glycerol export rather than uptake during hypo-osmotic shock [9]. However, when only non-fermentable carbon sources (glycerol, acetate, ethanol) are present, an active uptake system driven by a proton motive force ensures the uptake of glycerol [7,10]. Two multi-membrane-spanning proteins encoded by *GUP1* and *GUP2* (Glycerol Uptake Protein), were first identified as being involved in active glycerol uptake in *S. cerevisiae*[10]. However, in later studies Gup1p and 2p were proposed to have different roles than glycerol transport [11]. A screen for genes encoding membrane proteins involved in glycerol assimilation in *S. cerevisiae* identified a gene, *STL1*, involved in active glycerol uptake. Stl1p is localized in the plasma membrane, is glucose repressed and inactivated by growth in glucose [7,12]. Importantly, it was concluded that the protein is a member of the sugar transporter family and acts as a glycerol proton symporter [12]. Stl1p has also been shown to have a function in glycerol uptake in several other yeasts. It has been shown that in *Candida albicans* glycerol was actively transported into the cells by a proton symporter encoded by the *C. albicans STL1*[13]. It has also been reported that *S. cerevisiae* strains harboring the *STL1* gene from *D. hansenii* slightly improved their growth and doubling times on glycerol [14].

After glycerol is transported into the cells, two different routes have been identified in yeasts for further assimilation: a phosphorylation route and an oxidation route. Within the first pathway, glycerol is dissimilated by glycerol kinase encoded by *GUT1* and then by glycerol-3-phosphate dehydrogenase encoded by *GUT2*, which is located at the outer surface of the mitochondrial membrane [6]. Another pathway is catalyzed by glycerol dehydrogenase encoded by *GCY1* followed by dihydroxyacetone kinase encoded by *DAK1* and *DAK2*. In *Schizosaccharomyces pombe,* it has been reported that glycerol is utilized solely by this pathway [15,16]. In both pathways, glycerol is converted to dihydroxyacetone phosphate, which then enters glycolysis. The pathways involved in glycerol utilization differ in different yeasts. In some yeast strains, all four enzymes in both pathways are present, but only one pathway functions for glycerol dissimilation [4,6]. In *S. cerevisiae*, it has been shown that glycerol is degraded by the phosphorylation pathway and that mutants lacking one of the two genes are incapable of utilizing glycerol [17]. Although the fermentative pathway was also discovered to be present in *S. cerevisiae*[18], the function is unknown. The production of glycerol has two functions in *S. cerevisiae*: redox balance and protection against osmotic stress as a compatible solute (osmolyte) [6,19]. Glycerol is commonly produced in the cytosol of yeasts from the glycolytic intermediate dihydroxyacetone phosphate. This compound is converted into glycerol in two steps that are catalyzed by glycerol-3- phosphate dehydrogenase (Gpd) and glycerol-3-phosphatase (Gpp) respectively. Each of these enzymatic activities has two isoenzymes, Gpd1p and Gpd2p, Gpp1p and Gpp2p [20].

Although there have been several studies on glycerol metabolism in *S. cerevisiae*, many industrially used baker’s yeast strains and laboratory strains grow poorly on glycerol. For example, the specific growth rate of the well-studied lab strain *S. cerevisiae* CBS 8066 under aerobic conditions was found to be very low, 0.010 ± 0.002 h^-1^, during shake flask cultivations in minimal medium containing glycerol as sole carbon source [21]. Strategies based on adaptive evolution, and overexpression or knock out of the genes involved in glycerol metabolism have been performed with the aim of increasing the host strain’s growth on glycerol and production of different value-added products have been pursued [21,22]. By adaptive evolution, strains with growth rates of up to 0.2 h^-1^ have been achieved [21]. However, one possible rate-limiting step during glycerol metabolism, glycerol transport, was not addressed in those approaches [23].

In this study, a comparison of the genome sequences of *S. cerevisiae* and *P. tannophilus* has been performed to study the genes involved in glycerol transport and glycerol metabolism in these two organisms. Quantitative real-time PCR was performed to compare transcript levels during growth on glycerol. The genes involved in glycerol transport in *P. tannophilus* were heterologously expressed in a *S. cerevisiae STL1* knockout strain to assess their function and to evaluate whether they could possibly contribute as transporter genes to improve the growth of *S. cerevisiae* on glycerol.

## Results

### Identification of genes in glycerol metabolism and analysis of glycerol transporters in *P. tannophilus*

In order to find homologous genes related to glycerol transport and metabolism in *P. tannophilus*, the genome sequence was searched by BLAST with the sequences of genes known to have these functions in *S. cerevisiae* as queries. In *P. tannophilus*, two putative glycerol facilitator and two putative glycerol symporter genes were found and named *PtFPS1*, *PtFPS2*, *PtSTL1* and *PtSTL2*, respectively. In the glycerol consumption pathways, the genes with high similarity to *S. cerevisiae* were found and named *PtGUT1*, *PtGUT2*, *PtGCY1*, *PtGCY2*, *PtDAK* (Table 1). In the glycerol production pathways, the genes found were named *PtGPD* and *PtGPP*. It was noticed that the genes *PtFPS2* and *PtGUT1* were located close to each other in the *P. tannophilus* genome. With the aim of predicting the functions of the putative genes, bioinformatics tools Blastx and Blastp were used, and functional domain predictions were applied. The results are presented in Table 1.

**Table 1:** **Genes potentially involved in glycerol metabolism in *P. tannophilus***

**Annotated function**	**Gene name *S. cerevisiae***	**Gene name *P. tannophilus***	**Size (bp)**	**Identity/Similarity**	**Accession no.**
Transport					
Glycerol Facilitator	*ScFPS1*	*PtFPS1*	1170	35/49	[GenBank:JQ481631]
		*PtFSP2*	972	32/51	[GenBank:JQ481632]
Glycerol Symporter	*ScSTL1*	*PtSTL1*	1728	34/51	[GenBank:JQ481633]
		*PtSTL2*	1905	31/50	[GenBank:JQ481634]
Consumption					
Glycerol kinase	*ScGUT1*	*PtGUT1*	1848	53/67	[GenBank:JQ481635]
G3P dehydrogenase	*ScGUT2*	*PtGUT2*	1998	52/66	[GenBank:JQ481636]
Glycerol dehydrogenase	*ScGCY0031*	*PtGCY1*	936	54/73	[GenBank:JQ481637]
		*PtGCY2*	933	51/70	[GenBank:JQ481638]
Dihydroxyacetone kinase	*ScDAK1*	*PtDAK*	1767	42/58	[GenBank:JQ481639]
	*ScDAK2*				
Production					
G3P dehydrogenase	*ScGPD1*	*PtGPD*	1314	67/80	[GenBank:JQ481640]
	*ScGPD2*				
G3-phosphatase	*ScGPP1*	*PtGPP*	705	33/53	[GenBank:JQ481641]
	*ScGPP2*				

The potential genes were identified by performing a BLAST search with known *S. cerevisiae* genes as queries. Sequences were deposited in Genbank and the accession no. is listed in the table. Sizes given for *P. tannophilus* genes.

**Figure 1:**
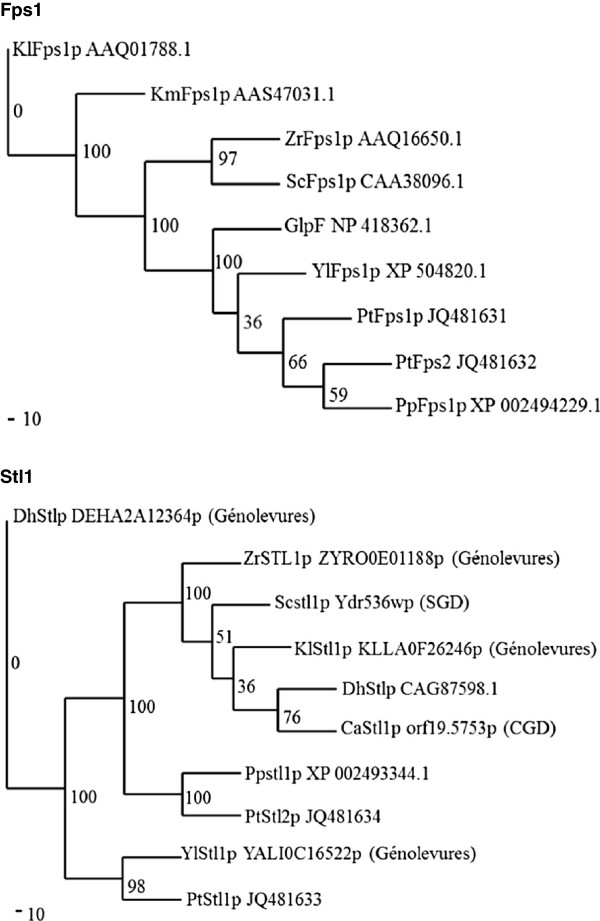
**Unrooted phylogenetic tree based on alignment of predicted protein sequences.** The tree was bootstrapped 1000 times. Accession numbers are presented after protein names. The origin of the protein sequences from databases other than GenBank are given in brackets after accession numbers. Ca, *Candida albicans;* Dh, *Debaryomyces hansenii*; Kl, *Kluyveromyces lactis*; Km, *Kluyveromyces marxianus*; Pp, *Pichia pastoris*; Pt, *Pachysolen tannophilus*; Sc, *Sacchromyces cerevisiae*; Yl, *Yarrowia lipolytica*; Zr, *Zygosaccharomyces rouxii;* GlpF*,* the aquaglyceroporin protein from. *E. coli*.

In order to investigate the phylogenetic relationship among putative glycerol transporters in *P. tannophilus* and their homologues in other yeast strains, an alignment was performed with Ptfps1p, Ptfps2p, Ptstl1p,Ptstl2p as well as with other predicted or published transporter proteins available from GenBank, Génolevures, SGD (Saccharomyces Genome Database) and CGD (Candida Genome Database). Unrooted phylogenetic trees are presented in Figure 1.

For *FPS1*, the lengths of PtFps1p, PtFps2p, PpFps1p, YaFps1p and GlpF were relatively short compared to other Fps1p proteins. PtFps1p was shown to be 54% identical to PpFps1p, 45% to YlFps1p, 35% to ScFps1p and only 31% to GlpF. PtFps2p was shown to be 60% identical to PpFps1p, 49% to YlFps1p, 34% to GlpF and only 32% to ScFps1p. PtFps1p and PtFps2p were grouped together with YlFps1p and PpFps1p, while they were in a separate branch than ScFps1p. For *STL1*, the identity between PtStl1p and ScStl1p was low, 34%, compared to the 56% identity observed between PtStl1p and YlPtStl1p.

### Quantitative real time PCR

**Figure 2:**
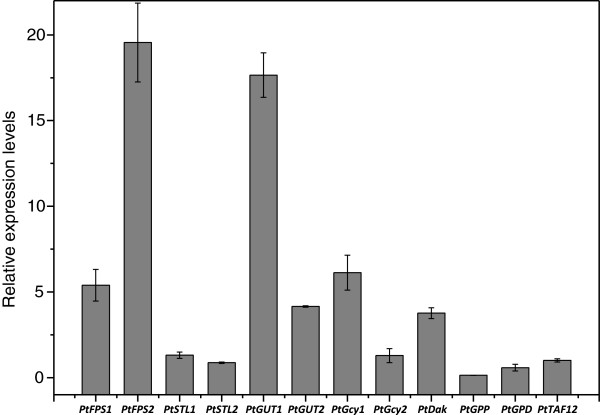
**Relative expression levels of glycerol metabolism related genes in *P. tannophilus *on glycerol compared to that on glucose.** The estimation of relative expression levels was based on 2^-ΔΔCT^, where ΔΔC_T_ = (C_T_ gene of interest - C_T_ internal control) sample A - (C_T_ gene of interest - C_T_ internal control) sample B. C_T_ represents the cycle number at which a sample reaches a predetermined threshold signal value for the specific target gene. All the experiments were performed in triplicate.

qPCR was used to compare the levels of transcripts during growth on glycerol to those on glucose as the sole carbon source. It was found that the genes involved in glycerol transport and assimilation *PtFPS1*, *PtFPS2*, *PtGUT1*, *PtGUT2*, *PtGCY1*, *PtDAK* were up-regulated on glycerol to different degrees compared to on glucose, while the genes involved with glycerol production *PtGPD* and *PtGPP* were down-regulated on glycerol (Figure 2). Among the genes *PtFPS2* and *PtGUT1* were most up-regulated, by a factor of 19.6 ± 2.3 and 17.6 ± 1.3, respectively, on glycerol compared to glucose. The transcript levels of *PtFPS1*, another putative facilitator, were up-regulated by a factor of 5.4 ± 0.9 on glycerol compared to glucose, while the putative glycerol symporter genes *PtSTL1* and *PtSTL2* were expressed almost at the same level on glycerol and glucose. *PtGUT2*, which is a putative mitochondrial glycerol phosphate ubiquinone oxidoreductase, was also up-regulated 4.2 ± 0.04 fold on glycerol.

### Performance of *S. cerevisiae stl1Δ* harboring different glycerol transporter genes

The glycerol symporter gene *ScSTL1*, which is responsible for glycerol transport in the absence of glucose, was knocked out. In order to validate the function of the glycerol transporters from *P. tannophilus*, all the predicted transporter genes from *P. tannophilus* were heterologously expressed in *S. cerevisiae stl1Δ*. Meanwhile, the glycerol facilitator Fps1p and glycerol symporter Stl1p in *S. cerevisiae* were also expressed in *S. cerevisiae stl1Δ.* To analyze the performance of the recombinant strains, growth in defined medium with 1% (v/v) glycerol as the sole carbon source was analyzed. The *stl1Δ* strain expressing different transporter genes *PtFPS1*, *PtFPS2*, *PtSTL1*, *PtSTL2*, *ScFPS1*, *ScSTL1* and the *S. cerevisiae* CENPK 113-5D strain were tested under aerobic conditions in shake flasks.

**Figure 3:**
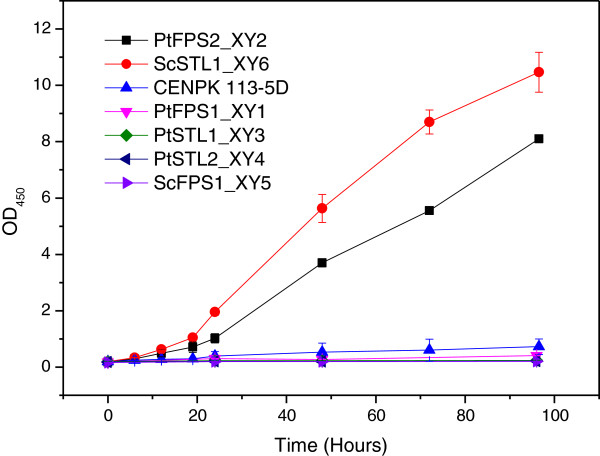
**Growth of recombinant *S. cerevisiae *strains expressing different glycerol transporter genes*.*** The growth was tested in defined medium containing 1% (v/v) glycerol as the sole carbon source at 30°C in agitated flasks. Results represent the mean of at least duplicate experiments.

The *stl1Δ* recombinant strain expressing the transporter gene *PtFPS2* from *P. tannophilus and ScSTL1* from *S. cerevisiae* showed growth on glycerol after 96 hours of cultivation, while the *stl1Δ* recombinant strain harbouring *PtFPS1*, *PtSTL1*, *PtSTL2 and ScFPS1* did not grow (Figure 3). The strains expressing *PtFPS2* and *ScSTL1* genes grew on glycerol with a μ_max_ of 0.07 ± 0.008 and 0.09 ± 0.005 and achieved a final OD of 8.1 and 10.5 at 96 hours cultivation, respectively. In comparison, the *S .cerevisiae* CENPK 113-5D strain grew very slowly with a μ_max_ of only 0.02 ± 0.004 on glycerol.

**Figure 4:**
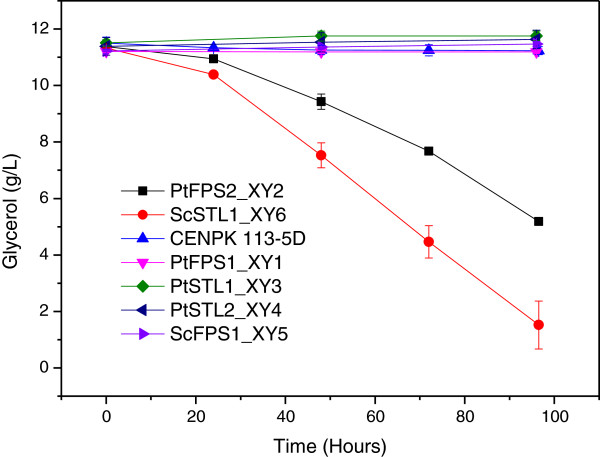
**Glycerol consumption of recombinant *S. cerevisiae *strains expressing different glycerol transporter genes.** The growth was tested in defined medium containing 1% (v/v) glycerol as the sole carbon source at 30°C in agitated shake flasks. Results represent the means of at least duplicate experiments.

In terms of glycerol consumption, the recombinant *stl1Δ* strains harboring the genes *PtFPS1*, *PtSTL1*, *PtSTL2* and *ScFPS1* did not consume glycerol even after 96 hours of cultivation. The *S .cerevisiae* CENPK 113-5D consumed only around 0.1 g glycerol. However, the recombinant *stl1Δ* strains harboring the genes *PtFPS2* and *ScSTL1* assimilated glycerol much faster than the other strains and the final glycerol consumed for the two strains was 6.2 ± 0.15 g/L and 9.8 ± 0.6 g/L glycerol, respectively (Figure 4). Although succinate was chosen as a buffer in all the experiments, it was not consumed by the strains.

## Discussion

With regard to glycerol transport, two glycerol facilitator homologues *PtFPS1*, *PtFPS2* and two glycerol symporter homologues *PtSTL1*, *PtSTL2* were found in the genome of *P. tannophilus*. Based on qPCR results, all the gene homologues identified to be involved in glycerol transport and assimilation were induced on glycerol as carbon source relative to the levels observed on glucose. The exceptions were the predicted glycerol symporters *PtSTL1* and *PtSTL2,* which were constitutively expressed on both glycerol and glucose as carbon sources. *PtFPS2* and *PtGUT1* (19.6 fold and 17.6 fold) were the two most strongly up-regulated genes, which might lead to the hypothesis that they are most relevant for glycerol assimilation in *P. tannophilus*. Moreover, the two genes are located closely together in the genome in agreement with a model where the two genes are co-regulated and contribute to the same overall process, i.e. glycerol assimilation. Next, we focused our efforts on understanding the function of the genes involved in glycerol transport as this function may be the rate limiting step for glycerol metabolism. In this paper the functions of the two types of glycerol transporters from *P. tannophilus* were addressed by transferring them individually to the well characterized yeast *S. cerevisiae.*

**Figure 5:**
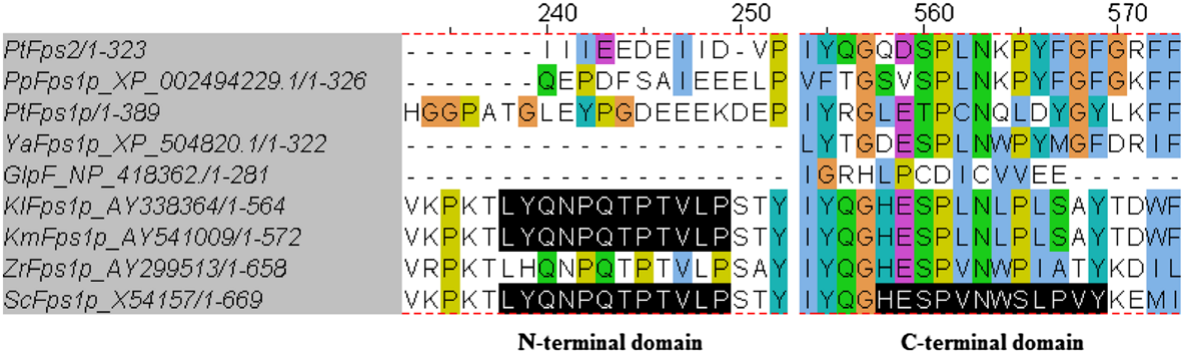
**Multiple sequence alignments with Fps1p from different yeast strains and *E. coli*.** The alignments were done by CLUSTALW2, showing the N-terminal and C-terminal extension domains of Fps1p.

The low affinity transporter Fps1p is a glycerol facilitator protein and belongs to the major intrinsic protein (MIP) family of channel proteins with six putative transmembrane domains (TMDs). Fps1p is responsible for transporting water, small molecules like glycerol, urea, NH_3_, CO_2_ or ions without consuming energy. The physiological role of the facilitator Fps1p in *S. cerevisiae* was previously described to be glycerol export rather than uptake during hypo-osmotic shock and the Fps1p channel closed and retained the glycerol inside the cells in response to hyperosmostic shock [9]. An N-terminal domain ^225^LYQNPQTPTVLP^236^ and a C-terminal domain ^535^HESPVNWSLPVY^546^ were found to have important roles in controlling Fps1p function. The N-terminus was required for closing of the channels and restricted transport through Fps1p. It was found that the rate of glycerol efflux was higher than that for uptake [9]. In agreement with this, we find in the present study that overexpression of *ScFPS1* does not suppress the glycerol transport defect of *stl1Δ* strains as they do not grow on glycerol. Surprisingly, overexpression of *PtFPS1* in *stl1*Δ strains allowed growth and assimilation of glycerol indicating that this homologous transporter is involved in glycerol uptake and recovers the *stl1*Δ strain growth defect. The similarities of PtFps1p and PtFps2p to ScFps1p are 35% and 32% respectively, but the homology is only restricted to the core of the protein with the six putative TMDs. ScFps1p (669 amino acid residues) is much longer than PtFps1p (389 residues) and PtFps2p (323 residues), the size of which is more similar to the *E. coli* GlpF (281 amino acid residues). The size difference is mainly due to the long hydrophilic N- and C-terminal domains of ScFps1p, which are absent in PtFps1p, PtFps2p and GlpF (Figure 5). By expressing *PtFPS2* in *S. cerevisiae stl1Δ* strains, the facilitators from *P. tannophilus* increased the glycerol influx and glycerol consumption, presumably due to improved glycerol transport ability. Previously it was also reported that the glycerol transport was increased approximately 2.5-fold in *S. cerevisiae* by introduction of the bacterial gene GlpF [9]. Therefore, we propose that in *P. tannophilus* the glycerol facilitator might function for glycerol influx rather than efflux.

The high-affinity transporter Stl1p was identified as the glycerol proton symporter in *S. cerevisiae*, which is a member of the sugar permease family of the major facilitator superfamily (MFS) [24]. It was demonstrated that the transcription of *STL1* gene was significantly induced with glycerol as the sole carbon source, and the *STL1* gene was subject to glucose repression based on microarray-based transcriptome analysis [25,26]. However, glycerol uptake by *STL1* from *C. albicans* was not affected by the carbon source and salt stress [13]. In *D. hansenii*, it was shown that the active glycerol transport system was constitutively expressed and not subject to glucose repression [27]. In agreement with these experiments, we showed that the glycerol symporter genes *PtSTL1* and *PtSTL2* in *P. tannophilus* are constitutively expressed on glycerol and glucose based on qPCR expression analysis. However, the presence of the *PtSTL1* and *PtSTL2* genes had no obvious effect on the physiology of *S. cerevisiae*, while the glycerol consumption and growth in strains that overexpressed *ScSTL1* was improved compared to *stl1Δ* strains. The symporter PtStl1p showed a low degree of sequence identities to ScStl1p with 34%. However, the PtStl1p from *P. tannophilus* exhibited 52% identity to the Stl1p from *D. hansenii* (DEHA2A12364p) and 56% to the Stl1p from *Yarrowia lipolytica* (YALI0C16522p). It has been reported that *Y. lipolytica* can grow on glycerol with a μ_max_ around 0.3 h^-1^[28] and *Pichia pastoris* can grow on glycerol with a μ_max_ 0.26 h^-1^[29]*. P. tannophilus* can grow on glycerol with μ_max_ around 0.29 h^-1^[2], while *S. cerevisiae* grows relatively slowly on glycerol with μ_max_ of 0.02 h^-1^ (CEN.PK 113-5D). Both the facilitator and symporter similarities among *P. tannophilus*, *Y. lipolytica* and *P. pastoris* were higher than that compared to *S. cerevisiae*. However, more protein sequence data with verified functions are needed to provide a definitive conclusion.

Since glycerol transport might be the rate-limiting step for glycerol utilization, heterologous expression of the glycerol transporters from yeasts which have relatively high growth rates on glycerol could be used as the approach for improving the efficiency of glycerol assimilation in *S. cerevisiae*. Improved glycerol transport has been demonstrated here with an increased glycerol consumption rate and growth rate under aerobic conditions with *S. cerevisiae*.

## Conclusions

The current study demonstrates the function of the glycerol transporters from *P. tannophilus*. Our studies open new possibilities for further improvement of glycerol fermentation in industrial yeast strains with heterologous expression of glycerol transporters from the glycerol utilizing *P. tannophilus*. This study thus proposes a possible route to development of glycerol-based bioprocesses in *S. cerevisiae*.

## Methods

### Strains and plasmids

The *P. tannophilus* strain used in this study was CBS4044. *S. cerevisiae* CEN.PK 113-5D was used for the construction of the *STL1* knockout strain. The plasmid PUG6 [30] was utilized as the template for amplifying of the *loxP-kanMX-loxP* cassette. The integrative USER vector pXI-5 [31] was used in this study for constructing the expression vectors. Plasmid pSP-G1 [32] was used as a template for amplifying the bidirectional promoter *TEF1*/*PGK1*. Plasmid pSH47 [30] was used for excision of the *kanMX* marker gene. All plasmids were propagated in *Escherichia coli* strain DH5α. All the plasmids and *S. cerevisiae* strains used in this study are listed in Table 2.

**Table 2:** Strains and plasmids used in this study

**Plasmid or strain**	**Relevant characteristics**	**Source or reference**
Plasmids		
PUG6	Plasmid with *loxP-kanMX-loxP* disruption cassette	[23]
pSP-G1	2μ-based URA3 plasmid with *TEF1*/*PGK1* promoter	[25]
pXI-5	Integrative USER vector, with reusable *URA3* Marker	[24]
pSH47	Centromeric plasmid, *URA3*, PGAL1-*Cre-*TCYC1	[23]
pXI-5-PtFPS1	pXI-5 with *TEF1*/*PGK1* promoter and *PtFPS1* gene	This study
pXI-5-PtFPS2	pXI-5 with *TEF1*/*PGK1* promoter and *PtFPS2* gene	This study
pXI-5-PtSTL1	pXI-5 with *TEF1*/*PGK1* promoter and *PtSTL1* gene	This study
pXI-5-PtSTL2	pXI-5 with *TEF1*/*PGK1* promoter and *PtSTL2* gene	This study
pXI-5-ScFPS1	pXI-5 with *TEF1*/*PGK1* promoter and *ScFPS1* gene	This study
pXI-5- ScSTL1	pXI-5 with *TEF1*/*PGK1* promoter and *ScSTL1* gene	This study
*S.cerevisiae* Strains		
CEN.PK 113-5D	*MAT****a****MAL2-8*^*c*^*SUC2 ura3-52*	Peter Kötter
*stl1Δ*	CEN.PK 113-5D *Stl1*Δ (490,1279 )::*loxP*	This study
PtFPS1_XY1	*stl1Δ* with plasmid pXI-5-PtFPS1	This study
PtFPS2_XY2	*stl1Δ* with plasmid pXI-5-PtFPS2	This study
PtSTL1_XY3	*stl1Δ* with plasmid pXI-5-PtSTL1	This study
PtSTL2_XY4	*stl1Δ* with plasmid pXI-5-PtSTL2	This study
ScFPS1_XY5	*stl1Δ* with plasmid pXI-5-ScFPS1	This study
ScSTL1_XY6	*stl1Δ* with plasmid pXI-5- ScSTL1	This study

### Medium and culture conditions

For quantitative real-time PCR (qPCR) experiments, *P. tannophilus* was grown at 30°C in defined medium, containing (per liter) 0.67 g yeast nitrogen base w/o amino acids, 10 g succinic acid and 6 g NaOH as buffer system (initial pH around 5.6), 2% (v/v) glycerol or 2% (w/v) glucose as the carbon source. Cells were harvested at mid exponential growth phase at the same optical density by centrifugation at 5000 g, 4°C for 5mins, resuspended in 1 ml ice cold ddH_2_O, and the cell pellet was stored at −80°C. *S. cerevisiae stl1Δ* harbouring different glycerol transporter genes were cultivated at 30°C for 96 hours in agitated flasks with defined medium, containing (per liter) 7.25 g synthetic complete (SC) powder, 10 g succinic acid and 6 g NaOH as buffer system, 1% (v/v) glycerol as the sole carbon source.

### Sequence retrieval and analysis

The genome sequence from *P. tannophilus* CBS4044 was obtained from previous work and is available in the EMBL database [3]. The sequences of genes involved in glycerol transport and metabolism in *S. cerevisiae FPS1*, *STL1*, *GUT1*, *GUT2*, *GCY1*, *Dak1/2*, *GPD1/2*, *GPP1/2* were used as queries in a BLAST search against the genome sequence of *P. tannophilus*. The genes with high similarities and high identities were listed as potential orthologous genes. Gene sequences were registered in GenBank at NCBI. The multiple sequence alignments with the amino acid sequences of *FPS1* and *STL1* transporters from different yeast strains and *E. coli* were performed by using ClustalW2 free program at PDBe. Phylogenetic analyses were performed by using PAUP* 4.0b10 [33]. Unweighted parsimony analysis was performed. Trees were inferred using the heuristic search option with tree bisection-reconnection branch swapping and 1000 random sequence additions. Maxtrees were unlimited, branches of zero length were collapsed and all multiple parsimonious trees were saved. Clade stability was assessed in a bootstrap analysis with 1000 replicates. Trees were visualized in Treeview [34].

### Quantitative real-time PCR

#### Total RNA isolation and cDNA synthesis

Total RNA was isolated from frozen cells using an RNeasy Mini Kit (QIAGEN, USA) according to the manufacturer’s protocol. The quantity and quality of the isolated RNA were measured by NanoDrop ND-1000. The total RNA was treated with DNaseI (Fermentas) prior to cDNA synthesis. Five μg total RNA were used to synthesize cDNA employing the RevertAid™ First Strand cDNA Synthesis kit (Fermentas) following the manufacturer’s recommendations using oligo(dT)_18_ primer. The cDNA was used as the template for quantitative real-time PCR for determining the transcript levels of the target genes under different growth conditions.

qPCR was performed on a Stratagene Mx3005P using the SYBR Green technology. The qPCR reaction mixture was prepared with 5 μl of 5 times dilution of cDNA as template, 10 μl SYBR Green master mix, 2 μl of 1 μM forward primer and 2 μl of 1 μM reverse primer and ddH_2_O to 20 μl. The PCR program for qPCR was as follows: 10 min of incubation at 95°C, followed by 40 cycles of 95°C for 30 s, 58°C for 30 s and 72°C for 30 s, and finally the temperature was increased from 55°C to 94°C to check for unspecific products. The number of fluorescence threshold cycles (Ct) was calculated with the set threshold value by using Mx3005P software. Results presented are mean values of three independent experiments. Suitable primer pairs for all the genes investigated were designed using the Primer Express software v3.0 (Applied Biosystems) software with the following parameters: product size 140 - 180 bp and melting temperature (Tm) 57-59°C. The primers used in this work are listed in Additional file 1. In addition, control reactions which include all components for qPCR, except for the reverse transcriptase, were performed to detect the genomic DNA contamination. The absence of products under these conditions meant the absence of enough genomic DNA for successful amplification.

The relative expression levels were calculated approximately based on 2^-ΔΔCT^, where ΔΔC_T_ = (C_T_ gene of interest - C_T_ internal control) sample A - (C_T_ gene of interest - C_T_ internal control) sample B. C_T_ represents the cycle number at which a sample reaches a predetermined threshold signal value for the specific target gene. Relative expression data were normalized to the relative expression value of the housekeeping gene *TAF12* in each sample, thus giving normalized relative expression for a target gene.

### *S. cerevisiae STL1* knockout strain construction

The construction of the *STL1* knockout strain was done through the *loxP-kanMX-loxP* /Cre recombinase system [30]. The primers for amplification of *loxP-kanMX-loxP* fragment from pUG6 were: KanMX_fw_STL1 (sequence 5^′^- AAAGCTGAAAATAGAGGGTTGCTGGTCAATTTAGAAGGTTCCACAATTGCCTGAAGCTTCGTACGCTG -3^′^) and KanMX_re_STL1 (sequence 5^′^- AAGCGTTTGTTGATGCACGAACTTTCATTGATGCAATTTCTGGTGGGTATCTAGTGGATCTGATATCAC -3^′^). The *loxP-kanMX-loxP* fragment with 50 bp homologous sequences in both ends to the gene *STL1*was transformed into CENPK-113-5D [35] for obtaining the knockout strain by selection colonies on YPD supplemented with 200 μg/ml Geneticin (G418). The resistant cells were transformed with pSH47 and selected on SC medium lacking uracil. Positive clones were purified and grown in liquid YPD overnight. The next day, the cells were washed once with distilled water and then incubated in YPG medium for 2 h in order to induce expression of the Cre recombinase. The culture medium was diluted and spread on YPD plates. The colonies were picked and restreaked on YPD plates supplemented with and without G418. Clones that were not able to grow on the G418 containing medium were the knockout strains. The knockout strains were streaked on 5-FOA (740 mg/L) plates to eliminate the pSH47 plasmid. Yeast extract-peptone-dextrose (YPD) medium, synthetic complete (SC) medium, and SC lacking specific amino acids were prepared as described previously [36].

### Constructs harboring different glycerol transporters

In this study, six expression plasmids carrying the glycerol transporter genes from *P. tannophilus* and *S.cerevisiae* were constructed. The USER vector pXI-5 was digested with AsiSI and then with the nicking endonuclease Nb.BsmI for making the AsiSI/Nb.BsmI USER cassettes [37]. The proofreading PfuX7 [38] was used for amplification of fragments *PtFPS1*, *PtFPS2*, *PtSTL1*, *PtSTL2, ScFPS1, ScSTL1* and bidirectional promoter *TEF1*/*PGK1* with appropriate USER tails for insertion into the designated USER cassette AsiSI/Nb.BsmI. Primers used are listed in Additional file 1. PCR was performed with PfuX7 DNA polymerase according to manufacturer’s instructions. DNA mixtures were prepared from purified digested vector, glycerol transporter gene, and bidirectional promoter *TEF1*/*PGK1*, 5× Phusion HF buffer (Fermentas), and 1 U of USER enzyme (Fermentas), adjusted to 10 μl by adding ddH_2_O. The mixture was incubated at 37°C for 20 min, followed by 25°C for 20 min. The 10 μl reaction mix was used directly to transform chemically competent *E. coli* DH5α cells. All the glycerol transporter genes were cloned under control of the *TEF1* promoter. Constructs were named as: pXI-5-PtFPS1, pXI-5-PtFPS2, pXI-5-PtSTL1, pXI-5-PtSTL2, pXI-5-ScFPS1, pXI-5-ScSTL1 (Table 2). The plasmids were verified by sequencing and digested with NotI (Fermentas) for 1 h at 37°C and then subjected to gel purification. Each linearized construct was homologously integrated into the *S. cerevisiae stl1Δ* genome by transformation; six yeast strain lines were constructed as summarized in Table 2. Transformants were plated onto SC-Ura for selection. The *S. cerevisiae* transformants were restreaked on SC-Ura plates for single colonies, and the desired homologous integration was checked by colony PCR. Sequences and descriptions of primers are presented in Additional file 1.

### Analytical methods

Growth was monitored by measuring OD_450nm_ with a Shimadzu UV mini-1240 spectrophotometer (Shimadzu, Japan). Samples were taken periodically from the flasks and filtered through a 0.22  μM syringe filter, and supernatants were preserved at −20°C for later HPLC analysis. Concentrations of the substrate glycerol in supernatants were measured by HPLC refractive index detector RID-10A using an Aminex87H column (Bio-Rad, USA). Separations were performed at 60°C, flow rate of 0.6 ml/min and 5 mM H_2_SO_4_ as mobile phase.

## Competing interests

The authors declare that they have no competing interests.

## Authors’ contributions

XL carried out the experimental work, analysis and drafted the manuscript. MW, supervised the experimental work and drafting of the manuscript, and has critically reviewed the text. UHM participated in the design and discussion of the experimental work and critical reviewing of the manuscript. All authors have read and approved the manuscript.

## Supplementary Material

Additional file 1 Primers used for Semi-quantitative RT-PCR and USER Cloning. Click here for file
