# Supplementary material for: Expression and functional studies of genes involved in transport and metabolism of glycerol in Pachysolen tannophilus
Source: Microb Cell Fact. 2013 Mar 21;12:27. doi: 10.1186/1475-2859-12-27 (PMC3610204; doi:10.1186/1475-2859-12-27)
Supplement: Additional file 1 — Primers used for Semi-quantitative RT-PCR and USER Cloning. [file 1475-2859-12-27-S1.docx]

**Primers used for Semi-quantitative RT-PCR and USER Cloning.**

| Primer Name | Sequence 5' to 3' |
| --- | --- |
| PtFPS1_qF | CTGCCGTTTACAGAGATTTT |
| PtFPS1_qR | AATATCTGCACCACCTTCGTA |
| PtFPS2_qF | GGTTTTGCTGTTATGATGGGTT |
| PtFPS2_qR | CAACATAAGCACCAATAAGACC |
| PtSTL1_qF | AATTAGGTTGTATGACTGGTGCATTT |
| PtSTL1_qR | TAGCAACTGCCAATTGTGCAA |
| PtSTL2_qF | TGGTTATGCAACTTCTGTTTGGA |
| PtSTL2_qR | AACCATCTTGGAGTTTCAACAATG |
| PtGcy1_qF | AACTGCCTTACAAAATGGGTACAG |
| PtGcy1_qR | TGCTTTAGCAACATCGTCTTGA |
| PtGcy2_qF | TGGTAACAAGATTCCGGCATT |
| PtGcy2_qR | ATTGCTTTGCCGACATCTTCT |
| PtDak_qF | GTAGTACTTTTGCTTCTCCTTCCACTAA |
| PtDak_qR | AACCTTCTGCTTTTGCTCTTTCA |
| PtGPD_qF | AGGTGTTTCAAAAATGAGTACGGTAT |
| PtGPD_qR | CCGGACCCTATCACTGCAA |
| PtGPP_qF | CAGTGGAAAGAGCTTGGAGTGT |
| PtGPP_qR | ACTCATCGGAAGCAGCATTG |
| PtGUT1_qF | CCAATTTGTGGTTGTTTAGGTG |
| PtGUT1_qR | CACCATGACGAGAGATCAAT |
| PtGUT2_qF | TCAAGAATGAATGTCGCGTT |
| PtGUT2_qR | CATCAGCACCAACCAAATATTC |
| PtTAF12_qF | CAAACCTTCATTACCACTGCC |
| PtTAF12_qR | GTTGCCATCTCCTTCATCG |
| TEF1_F_U | AGCAGTGAUTTGTAATTAAAACTTAGATTAGATTGC |
| PGK1_R_U | CACGCGAUTTGTTTTATATTTGTTGTAAAAAGTAGA |
| PtFPS1_F_U | ATCACTGCUATGTCAAATTCATCCGGGAAC |
| PtFPS1_R_U | CGTGCGAUTTAAGTAGAGTCAACTTCTTTTTCAAG |
| PtFPS2_F_U | ATCACTGCUATGCAAATAGAGAATGTTCAAGGA |
| PtFPS2_R_U | CGTGCGAUTTAGTATTGATTTTCTACGTCTTTTGTT |
| PtSTL1_F_U | ATCACTGCUATGTTCAAAAAAATCGATAAAATTG |
| PtSTL1_R_U | CGTGCGAAUTCACTCTTTCTTTTCGGGTT |
| PtSTL2_F_U | ATCACTGCUATGGATTCGAATATAGATGATACAG |
| PtSTL2_R_U | CGTGCGAUCTAATGACGACCGCTATTG |
| ScFPS1_F_U | ATCACTGCUATGAGTAATCCTCAAAAAGCTC |
| ScFPS1_R_U | CGTGCGAUTCATGTTACCTTCTTAGCATTAC |
| ScSTL1_F_U | ATCACTGCUATGAAGGATTTAAAATTATCGAATTTC |
| ScSTL1_R_U | CGTGCGAUTCAACCCTCAAAATTTGCT |
